# SLC1A5 is a key regulator of glutamine metabolism and a prognostic marker for aggressive luminal breast cancer

**DOI:** 10.1038/s41598-025-87292-1

**Published:** 2025-01-22

**Authors:** Lutfi H. Alfarsi, Rokaya El Ansari, Busra Erkan, Ali Fakroun, Madeleine L. Craze, Mohammed A. Aleskandarany, Kiu Wai Cheng, Ian O. Ellis, Emad A. Rakha, Andrew R. Green

**Affiliations:** 1 Nottingham Breast Cancer Research Centre, Academic Unit of Translational Medical Sciences, School of Medicine, University of Nottingham, University of Nottingham Biodiscovery Institute, University Park, Nottingham, NG7 2RD England; 2 Cellular Pathology, Nottingham University Hospitals NHS Trust, Hucknall Road, Nottingham, NG5 1PB England

**Keywords:** SLC1A5, Breast cancer, Prognosis, Tumour marker, Cancer, Breast cancer, Cancer metabolism

## Abstract

Cancer cells exhibit altered metabolism, often relying on glutamine (Gln) for growth. Breast cancer (BC) is a heterogeneous disease with varying clinical outcomes. We investigated the role of the amino acid transporter SLC1A5 (ASCT2) and its association with BC subtypes and patient outcomes. In large BC cohorts, *SLC1A5* mRNA (n = 9488) and SLC1A5 protein (n = 1274) levels were assessed and correlated their expression with clinicopathological features, molecular subtypes, and patient outcomes. In vitro* SLC1A5* knockdown and inhibition studies in luminal BC cell lines (ZR-75-1 and HCC1500) were used to further explore the role of SLC1A5 in Gln metabolism. Statistical analysis was performed using chi-squared tests, ANOVA, Spearman’s correlation, Kaplan–Meier analysis, and Cox regression. *SLC1A5 mRNA* and SLC1A5 protein expression were strongly correlated in luminal B, HER2 + and triple-negative BC (TNBC). Both high *SLC1A5 mRNA* and SLC1A5 protein expression were associated with larger tumour size, higher grade, and positive axillary lymph node metastases (P < 0.01). Importantly, high SLC1A5 expression correlated with poor BC-specific survival specifically in the highly proliferative luminal subtype (P < 0.001). Furthermore, *SLC1A5* knockdown by siRNA or GPNA inhibition significantly reduced cell proliferation and glutamine uptake in ZR-75-1 cells. Our findings suggest SLC1A5 plays a key role in the aggressive luminal BC subtype and represents a potential therapeutic target. Further research is needed to explore SLC1A5 function in luminal BC and its association with Gln metabolism pathways.

**Supplementary Information:**

The online version contains supplementary material available at 10.1038/s41598-025-87292-1.

## Introduction

Deregulation of metabolic pathways has been readily accepted as one of the revised hallmarks of cancer, where cancer cells are able to regulate their metabolism to provide the energy and cellular building blocks required for growth^1^. Many cancer cells are highly reliant on amino acids for their growth, where endogenous synthesis may not provide rapidly proliferating cells with sufficient nutrients for nuclear biosynthesis. There is also increasing evidence that oncogenes and/or tumour suppressor genes can reprogramme tumour cell metabolism, including through direct regulation of the proline–glutamine regulatory axis by MYC and p53^2–4^. This axis is the most important metabolic pathway in tumours, after which glucose, primarily glutamine, is used to replenish the tricarboxylic acid (TCA) cycle and supplies carbon and nitrogen for the synthesis of nucleotides, amino acids and glutathione. Indeed, some solid tumours exhibit glutamine-dependent cell growth or “glutamine addiction”^5^.

L-Glutamine (Gln) is a nonessential amino acid synthesised from glutamate and ammonia by glutamine synthetase (GS). Its utilisation, via reductive carboxylation, is necessary for sustained proliferation/survival and is linked with resistance to certain drugs^6^. A further role for Gln in cancer cell protein translation stems from observations that a master regulator of protein translation, rapamycin complex 1 (mTORC1), which regulates cell growth and protein translation, is also responsive to glutamine levels^7,8^. In BC, highly proliferative high-grade tumors, such as those in the triple negative (TN) class, have higher levels of glutamate and glutaminase (GLS) together with lower levels of Gln than low-grade tumours and normal breast epithelium^9–13^. The metabolic profiles of BC show that glutaminolysis metabolism is a key pathway for discriminating between TN and luminal/oestrogen receptor-positive (ER +) tumours^14^.

Solute carrier family 1 member 5 (SLC1A5)/ASC amino acid transporter 2 (ASCT2) is a cell surface sodium-dependent transporter that regulates the uptake of neutral amino acids, including Gln^15,16^. Inhibiting SLC1A5 resulted in reduced cellular proliferation in several cancer types, including non-small cell lung cancer^17,18^, renal cell carcinoma^19^, pancreatic carcinoma^20^, prostate carcinoma^21^ and melanoma^22^. In BC, it is reported to be highly expressed in HER2-positive but not in luminal A tumours^10^, although the uptake of Gln is only required for basal-like TNBC to sustain mTORC1 signalling^8^. SLC1A5 is also upregulated by MYC and downregulated by retinoblastoma (Rb)^5,23^.

With renewed interest in oncometabolism, metabolic enzymes are increasingly being targeted to improve therapeutic efficacy and reduce resistance. We therefore hypothesised that the proline-Gln axis is a key metabolic pathway regulated by MYC in BC, particularly because we have shown that proline dehydrogenase (PRODH) is downregulated^2^. This pathway could be used as a potential therapeutic target, particularly because the pleotropic MYC has thus far proven ineffective as a target. Therefore, the aim of this study was to assess *SLC1A5* gene copy number and its expression at both the *mRNA* and protein levels in large and well-characterised annotated cohorts of BC patients combined with in vitro approaches to determine its biological, clinicopathological and prognostic value in different molecular classes, with particular interest in highly proliferative aggressive subgroups.

## Materials and methods

### *SLC1A5* genomic and transcriptomic analysis

*SLC1A5* gene expression was evaluated in a cohort of 1,980 BC samples using the Molecular Taxonomy of BC International Consortium (METABRIC) cohort^24^. RNA from fresh frozen tumours was subjected to transcriptional profiling using the Illumina HT-12 v3 platform, and the data were preprocessed and normalised as described previously^24^. In this cohort, patients with ER + and/or lymph node-negative tumours did not receive adjuvant chemotherapy, while those with ER-negative and/or lymph node-positive tumours received adjuvant chemotherapy. The relationships between copy number (CN) aberrations, both gains and losses, of *SLC1A5* and *TP53* mutations and *SLC1A5 mRNA* expression and patient outcome were investigated. The clinicopathological parameters for this dataset are summarised in Supplementary Table 1.

For the external validation of *SLC1A5 mRNA* expression, the BC Gene-Expression Miner v5.0 (n = 4712) (http://bcgenex.centregauducheau.fr) and Kaplan‒Meier plotter (n = 2,796) (http://kmplot.com) datasets were used.

### SLC1A5 proteomic analysis

#### Patient cohort

Immunohistochemistry (IHC) was conducted using a large cohort of patients comprising a well-characterised consecutive series of early-stage (TNM stage I-III excluding T3 and T4 tumours) sporadic primary operable invasive BC. Patients (aged ≤ 70 years) who presented at Nottingham City Hospital between 1989 and 1998 (n = 1274) and were managed in accordance with a uniform protocol were enrolled in the Nottingham Tenovus Primary Breast Carcinoma Series. Patients’ clinical history, tumour characteristics, and information on therapy and outcomes were prospectively collected. Outcome data were collected on a prospective basis and included development and time to distant metastasis (DM) and BC-specific survival (BCSS). The BCSS was defined as the time (in months) from the date of primary surgery to the date of BC-related death. DM-free survival (DMFS) was defined as the time (in months) from the date of primary surgery to the appearance of DM. The clinicopathological parameters of this cohort of patients are summarised in Supplementary Table 1.

#### Tissue microarrays (TMAs) and IHC

Tumour samples (0.6 mm cores) were arrayed as previously described^25^. Immunohistochemical staining was performed on 4 μm sections using a Novolink polymer detection system (RE7150-K, Leica Biosystems, UK). Briefly, tissue slides were deparaffinised with xylene and rehydrated through 3 changes of alcohol. Heat-induced antigen epitope retrieval was performed in citrate buffer (pH 6.0) for 20 min using a microwave oven (Whirpool JT359 Jet Chef 1000 W). Endogenous peroxidase activity was blocked with a peroxidase block for 5 min. The slides were washed with Tris-buffered saline (TBS, pH 7.6), followed by the application of a protein block for 5 min. Following another TBS wash, a mouse monoclonal primary antibody against SLC1A5 (HPA035240, Sigma‒Aldrich, UK) diluted at 1:100 in Leica antibody diluent (RE7133 Leica, Biosysytems, UK) was applied, and the membrane was incubated for 30 min. The slides were washed with TBS, incubated with postprimary block for 30 min, and then washed with TBS. Novolink polymer was applied for 30 min. DAB working solution composed of 1:20 DAB chromogen in DAB substrate buffer was prepared and applied for 5 min. Slides were counterstained with Novolink haematoxylin for 6 min, dehydrated and coverslipped. Negative (omission of the primary antibody) and positive controls were included according to the manufacturer’s datasheet for each antibody.

The stained TMA sections were scored using high-resolution digital images (NanoZoomer; Hamamatsu Photonics, Welwyn Garden City, UK) at × 20 magnification. Assessment of SLC1A5 staining was based on a semiquantitative assessment of core digital images using a modified histochemical score (H-score), which includes an assessment of both the intensity of staining and the percentage of stained cells [29]. TMA cores were only assessed if the tumour burden was > 15%^26,27^.

Immunohistochemical staining and dichotomisation of the other biomarkers included in this study were performed according to previous publications^2,25,28–38^. ER and progesterone receptor (PR) positivity was defined as ≥ 1% staining. The immunoreactivity of HER2 in the TMA cores was scored using standard HercepTest guidelines (Dako). Chromogenic in situ hybridisation (CISH) was used to quantify HER2 gene amplification in borderline cases using the HER2 FISH pharmDx™ plus HER2 CISH pharmDx™ kit (Dako) and was assessed according to the American Society of Clinical Oncology guidelines. BC molecular subtypes were defined based on the IHC profile as follows: ER + /HER2- low proliferation (Ki67 < 10%); ER + /HER2- high proliferation (Ki67 ≥ 10%); and the HER2 + class, HER2 + regardless of the ER status and triple negative (TN) subtype, ER-, PR- and HER2-. The basal phenotype was defined as those tumours expressing cytokeratin (Ck) 5/6 and/or Ck14 and/or Ck17.

#### Cell culture

The luminal BC cell lines MDA-MB-175, ZR-75-1 (ER + /PR-/HER2-), T47D, MCF7, and HCC1500 (ER + /PR + /HER2-) were obtained from the American Type Culture Collection (Rockville, MD, USA). The cells were cultured in Roswell Park Memorial Institute (RPMI-1640) medium (Sigma‒Aldrich, UK) supplemented with 10% foetal bovine serum (Sigma‒Aldrich, UK). Mycoplasma testing was carried out on a routine basis using the MycoAlert Detection Kit (R&D Systems).

#### SLC1A5 inhibition and knockdown

SLC1A5 inhibition was achieved in ZR-75-1 and HCC1500 cells cultured in 96-well plates at a density of 1 × 10^3^ cells per well. Cultures were treated with the SLC1A5 inhibitor gamma-l-glutamyl-p-nitroanilide (GPNA) (1 mM; Sigma‒Aldrich, UK).

*SLC1A5* knockdown in ZR-75-1 and HCC1500 cells was performed via transfection of 2 × 10^5^ cells/well in 6-well plates via the reverse transfection method with 100 pmol of siRNA (Thermo Fisher Scientific, UK) and 5 µl of Lipofectamine RNAiMAX (Thermo Fisher Scientific, UK) according to the manufacturer’s protocol. The sequence of the antisense siRNA used was 5’-AAAGAGUAAACCCACAUCCtc-3’. Scrambled siRNA was used alongside the experiment as a negative control. *SLC1A5* knockdown was confirmed by Western blotting. Functional assays were carried out in the transfected and control cells 24 h after transfection and in cultured cells with or without GPNA.

#### Cell proliferation assay

Cells were seeded at a density of 1 × 10^3^ cells per well in a 96-well plate in triplicate. MTS assays were conducted every day for 4 days to assess cell growth according to the manufacturer’s instructions (MTS CellTiter 96 Aqueous One Solution) (Promega, UK). The absorbance was recorded at 490 nm using a microplate reader (TECAN Infinite F50). The background absorbance from the empty wells was subtracted from that of the sample wells.

#### Glutamine uptake assay

A total of 5 × 10^4^ cells were incubated with ^3^H-L-glutamine (250 µci; Perkin Elmer) in glutamine-free media (Sigma‒Aldrich, UK) for 30 min at 37 °C in the presence/absence of transfection or inhibitor. The cells were washed twice with Dulbecco’s phosphate-buffered saline (Sigma‒Aldrich, UK). The cell pellets were resuspended in glutamine-free media and loaded onto Luma plates (Perkin Elmer). Radioactivity was measured using a scintillation counter (PerkinElmer, USA).

#### Western blotting

Cells were harvested and lysed in lysis buffer. The samples were subjected to SDS‒polyacrylamide gel electrophoresis (PAGE) and transferred to PVDF membranes (Immobilon-FL). A 1:250 dilution of the primary SLC1A5 antibody and a 1:5000 dilution of the mouse monoclonal anti-β-actin primary antibody (A5441, Sigma‒Aldrich, UK) were used as loading controls. IRDye 800CW donkey anti-rabbit fluorescent secondary antibody (1:15,000 dilution) and IRDye 600RD donkey anti-mouse fluorescent secondary antibody (926–32,213 and 926–68,072, LI-COR Biosciences, UK) were used. A PageRuler Plus Prestained Protein Ladder (26,619, Thermo Fisher Scientific, UK) was used. An Odyssey Fc with Image Studio 4.0 was used to visualise the bands (LI-COR Biosciences).

### Statistical analysis

Statistical analysis was performed using SPSS software (SPSS Inc., Chicago, IL, USA). The chi-squared test was used to evaluate the significance of associations with clinicopathological parameters. One-way ANOVA with the post hoc Tukey multiple comparison test and Spearman’s correlation coefficient were used for continuous data. The Kaplan–Meier method and Cox proportional hazards model were used to evaluate the prognostic results. Dichotomisation of *SLC1A5* mRNA and SLC1A5 protein expression was performed using X-tile software (version 3.6.1, Yale University, USA) based on outcome prediction. A P value < 0.05 was considered to indicate statistical significance.

## Results

### *SLC1A5* copy number and mRNA expression in breast cancer

**Fig. 1:**
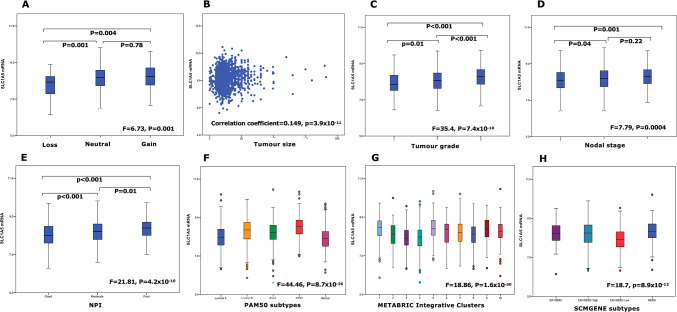
*SLC1A5* mRNA expression and its association with invasive breast cancer clinicopathological parameters and molecular subtypes in the METABRIC cohort: (**A**) SLC1A5 copy number variation, (**B**) tumour size, (**C**) tumour grade, (**D**) lymph node stage, (**E**) Nottingham Prognostic Index (NPI), F) PAM50 subtypes (**G**) METABRIC Integrative Clusters, (**H**) SMCGENE subtypes.

*SLC1A5* CN gains were observed in 3% and CN loss in 2% of invasive BC patients, whereas high *SLC1A5* mRNA expression was observed in 61.4% of the tumours. Those with a CN loss of the *SLC1A5* gene exhibited significantly lower *SLC1A5* mRNA expression (P = 0.001; Fig. 1A). Conversely, tumours with CN gain of *SLC1A5* did not show a corresponding increase in *SLC1A5* mRNA levels.

### Clinicopathological parameters and molecular subtypes

High *SLC1A5 mRNA* expression was significantly associated with larger tumour size, higher grade and nodal stage together with poor NPI (all P < 0.001, Fig. 1B–E). These associations were confirmed using bc-GenExMiner (Supplementary Fig. 1).

High *SLC1A5 mRNA* expression was significantly associated with ER-negative, PR-negative, and HER2-positive tumours together with TN tumours (all P < 0.01, Table 1). When comparing the levels of *SLC1A5 mRNA* expression in the intrinsic (PAM50) subtypes [39], high expression was observed in basal, HER2-enriched and luminal B tumours (P < 0.001, Fig. 1F). Similarly, within the METABRIC Integrative Clusters, high *SLC1A5 mRNA* expression was associated with clusters 1 (luminal B subgroup), 5 (ERBB2 amplified), 9 (luminal B subgroup) and 10 (triple negative/basal-like) (P < 0.001, Fig. 1G). In the SCMGENE subtypes, there was greater expression of *SLC1A5 mRNA* in the ER + /HER2- high proliferation class (luminal B) than in the ER + /HER2- low proliferation class (luminal A) (P < 0.001, Fig. 1H). The associations of *SLC1A5 mRNA* with ER- and PR- tumours, as well as with HER2 + basal and luminal B tumours (PAM50), were confirmed using bc-GenExMiner (Supplementary Fig. 1). High *SLC1A5* mRNA expression was detected in the luminal androgen receptor (LAR) subtype compared with the basal-like immunosuppressed (BLIS), basal-like immune-activated (BLIA), and mesenchymal (MES) TN subtypes (Supplementary Fig. 1H).

**Table 1:** Association of SLC1A5 expression at *mRNA* and protein levels and other molecular biomarkers in breast cancer.

SLC1A5								
	mRNA				Protein			
	Low n (%)	High n (%)	χ2 (p-value)	Adjusted p-value	Low n (%)	High n (%)	χ2 (p-value)	Adjusted p-value
ER								
Negative	123 (26.1)	348 (73.9)	40.2 (2.3 × 10^–10^)	** < 0.0001**	107 (18.0)	488 (82.0)	120.0 (6.2 × 10^–28^)	** < 0.0001**
Positive	639 (42.4)	868 (57.6)			918 (42.5)	1242 (57.5)		
PR								
Negative	298 (31.8)	640 (68.2)	34.4 (4.6 × 10^–9^)	** < 0.0001**	306 (28.0)	785 (72.0)	61.37 (4.7 × 10^–15^)	** < 0.0001**
Positive	464 (44.6)	576 (55.4)			691 (42.9)	921 (57.1)		
HER2								
Negative	710 (41.0)	1023 (59.0)	35.4 (2.8 × 10^–9^)	** < 0.0001**	929 (39.5)	1424 (60.5)	45.3 (1.6 × 10^–11^)	** < 0.0001**
Positive	52 (21.2)	193 (78.8)			73 (20.9)	277 (79.1)		
Triple negative								
No	661 (39.8)	999 (60.2)	7.3 (0.007)	**0.008**	942 (40.8)	1367 (59.2)	83.1 (7.7 × 10^–20^)	** < 0.0001**
Yes	101 (31.8)	217 (68.2)			74 (17.5)	349 (82.5)		
TP53 mutation								
Wild-type	316 (44.1)	401 (55.9)	22.3 (0.000002)	** < 0.0001**	N/A			
Mutation	19 (19.2)	80 (80.8)						
P53 protein								
Negative	N/A				344 (40.6)	503 (59.4)	32.7 (1.1 × 10^–8^ )	** < 0.0001**
Positive					86 (23.5)	280 (76.5)		

Significant values are in [bold].

High *SLC1A5* mRNA expression in breast tumors correlated with a high frequency of *TP53* mutations (P < 0.001, Table 1). Gene CN analysis revealed that luminal B tumours had the greatest proportion of *SLC1A5* CN loss (P = 0.02, Supplementary Table 2), whereas no significant association was detected between *SLC1A5* gain and the intrinsic BC subtypes (P > 0.05, Supplementary Table 2).

### Regulatory and amino acid transporter genes

The correlation between *SLC1A5 mRNA* and other related genes was investigated (Table 2). The genes were selected based on previous publications, as they are either regulatory genes or others that share the biological functions of amino acid transporters, which are primarily focused on glutamine transport and glutamine metabolism.

**Table 2:** Correlation of *SLC1A5 mRNA* expression with the expression of other related genes in primary invasive breast cancer using the METABRIC cohort.

	*SLC1A5 mRNA*									
	All cases (n = 1,980)		Luminal A (n = 368)		Luminal B (n = 367)		HER2 + (n = 110)		Triple negative (n = 150)	
Correlation Coefficient (p-value)						Adjusted p-value				
Regulatory and other associated genes										
MYC	− 0.126 (1.6 × 10^–8^)	** < 0.0001**	− 0.179 (0.001)	**0.01**	− 0.041 (0.438)	5.1	− 0.263 (0.006)	0.13	− 0.031 (0.709)	3.5
Rb	− 0.23 (2.4 × 10^–25^)	** < 0.0001**	− 0.154 (0.00003)	**0.0007**	− 0.168 (0.0001)	**0.002**	− 0.285 (0.000007)	**0.0002**	− 0.305 (1.7 × 10^–8^)	** < 0.0001**
PIK3CA	0.196 (1.5 × 10^–18^)	** < 0.0001**	0.208 (1.7 × 10^–8^)	** < 0.0001**	0.134 (0.003)	**0.007**	0.126 (0.05)	0.56	0.236 (0.00001)	**0.0003**
AKT1	0.148 (3.5 × 10^–11^)	** < 0.0001**	0.230 (0.000008)	**0.0002**	0.006 (0.903)	2.7	0.189 (0.048)	0.48	0.204 (0.012)	0.16
RAF1	0.136 (1.1 × 10^–9^)	** < 0.0001**	0.103 (0.006)	0.11	0.098 (0.03)	0.63	0.134 (0.03)	0.45	0.112 (0.04)	0.60
BRAF	0.108 (0.000001)	** < 0.0001**	0.08 (0.01)	0.12	− 0.042 (0.357)	4.9	0.09 (0.162)	1.41	0.215 (0.00008)	**0.001**
KRAS	0.127 (1.4 × 10^–8^)	** < 0.0001**	0.07 (0.03)	0.27	0.119 (0.009)	0.19	− 0.04 (0.49)	1.98	0.149 (0.007)	0.13
EPHA2	0.068 (0.002)	**0.02**	0.109 (0.003)	0.30	0.001 (0.987)	0.98	0.114 (0.07)	0.70	0.08 (0.13)	1.56
ATF4	0.273 (4.3 × 10^–35^)	** < 0.0001**	0.106 (0.042)	0.33	0.215 (0.00004)	**0.001**	0.445 (0.000001)	** < 0.0001**	0.370 (0.000003)	**0.0001**
Enzymes involved in glutamine metabolism										
GLS	− 0.087 (0.0001)	**0.001**	− 0.111 (0.033)	0.30	− 0.064 (0.219)	3.7	− 0.183 (0.056)	0.60	0.076 (0.357)	1.58
ALDH4A1	0.042 (0.064)	0.30	− 0.144 (0.006)	0.11	− 0.037 (0.482)	5.2	0.220 (0.021)	0.34	0.055 (0.505)	3.04
PRODH	0.052 (0.021)	0.18	0.179 (0.001)	**0.01**	− 0.014 (0.795)	4.7	0.222 (0.020)	0.36	0.071 (0.385)	2.10
PYCR1	0.335 (4.7 × 10^–53^)	** < 0.0001**	0.267 (1.9 × 10^–7^)	** < 0.0001**	0.245 (0.000002)	**0.0001**	0.445 (0.000001)	** < 0.0001**	0.362 (0.000005)	**0.0001**
ALDH18A1	0.164 (2.4 × 10^–13^)	** < 0.0001**	0.227 (0.00001)	**0.0002**	0.057 (0.278)	4.3	0.278 (0.003)	0.07	0.217 (0.008)	0.14
GLUL	0.016 (0.478)	1.40	0.158 (0.002)	**0.02**	− 0.065 (0.214)	3.5	− 0.072 (0.454)	1.62	− 0.067 (0.415)	2.52
GLUD1	0.134 (2.5 × 10^–9^)	** < 0.0001**	0.278 (5.8 × 10^–8^)	** < 0.0001**	0.087 (0.096)	0.80	0.116 (0.228)	1.45	0.076 (0.358)	2.00
Amino acid transporters										
SLC7A5	0.29 (4.5 × 10^–41^)	** < 0.0001**	0.170 (0.000005)	**0.0001**	0.150 (0.001)	**0.02**	0.208 (0.001)	**0.02**	0.25 (0.000002)	** < 0.0001**
SLC3A2	− 0.098 (0.00001)	**0.0001**	− 0.188 (0.0003)	**0.006**	− 0.077 (0.142)	2.6	− 0.209 (0.028)	0.38	− 0.129 (0.115)	1.43
SLC6A19	0.01 (0.47)	0.94	0.02 (0.44)	1.3	0.01 (0.74)	5.9	− 0.11 (0.03)	0.46	0.04 (0.50)	3.42
SLC7A6	0.129 (8.8 × 10^–9^)	** < 0.0001**	0.03 (0.29)	1.4	0.01 (0.76)	5.3	0.137 (0.01)	0.20	0.18 (0.004)	0.08
SLC7A7	− 0.01 (0.40)	1.60	− 0.104 (0.005)	0.10	− 0.04 (0.28)	4.2	− 0.04 (0.39)	1.60	− 0.21 (0.001)	**0.02**
SLC7A8	− 0.07 (0.001)	**0.01**	0.06 (0.09)	0.54	0.007 (0.88)	3.2	− 0.03 (0.62)	1.80	− 0.20 (0.0002)	**0.003**
SLC7A9	0.057 (0.01)	0.08	0.06 (0.101)	0.70	− 0.01 (0.72)	6.4	0.15 (0.006)	0.13	0.05 (0.38)	2.50
SLC38A1	0.066 (0.003)	**0.03**	-0.028 (0.596)	1.1	0.042 (0.427)	5.4	0.105 (0.275)	1.54	0.227 (0.005)	0.10
SLC38A2	0.18 (1.5 × 10^–16^)	** < 0.0001**	0.17 (0.000004)	**0.0001**	0.14 (0.002)	**0.02**	0.19 (0.002)	**0.02**	0.17 (0.002)	**0.04**
SLC38A3	0.069 (0.002)	**0.02**	0.053 (0.307)	1.2	− 0.020 (0.704)	6.0	0.121 (0.206)	1.44	0.128 (0.119)	1.45
SLC38A5	0.04 (0.03)	0.18	− 0.05 (0.11)	0.6	0.01 (0.79)	4.7	0.10 (0.06)	0.65	0.05 (0.42)	2.87
SLC38A7	0.241 (1.2 × 10^–27^)	** < 0.0001**	0.203 (4.0 × 10^–8^)	** < 0.0001**	0.145 (0.001)	**0.03**	0.168 (0.002)	0.05	0.31 (0.000001)	** < 0.0001**
SLC38A8	0.01 (0.48)	0.48	0.01 (0.79)	0.79	0.005 (0.91)	1.8	− 0.03 (0.47)	1.80	− 0.01 (0.79)	3.85

There was a significant correlation between *SLC1A5 mRNA* expression and the expression of all regulatory genes that were previously identified in the literature, including *MYC*, *Rb*, *ATF4*, *PIK3CA*, *EphA2* and genes involved in the MAPK pathway (*RAF1*, *BRAF* and *KRAS*) (P < 0.05, Table 2). While many regulatory genes displayed a positive correlation with *SLC1A5*, an inverse relationship was observed between *MYC* and *Rb*, with the latter showing a consistent negative correlation across all BC subtypes (P < 0.01). However, the negative correlation between *MYC* and *SLC1A5* expression was significant only for luminal A tumours (P = 0.01) and not for the other subtypes (P > 0.05). *PIK3CA* was the only gene that showed a positive relationship in all BC subtypes, excluding HER2 + tumours (P < 0.01 and P > 0.05).

**Fig. 2:**
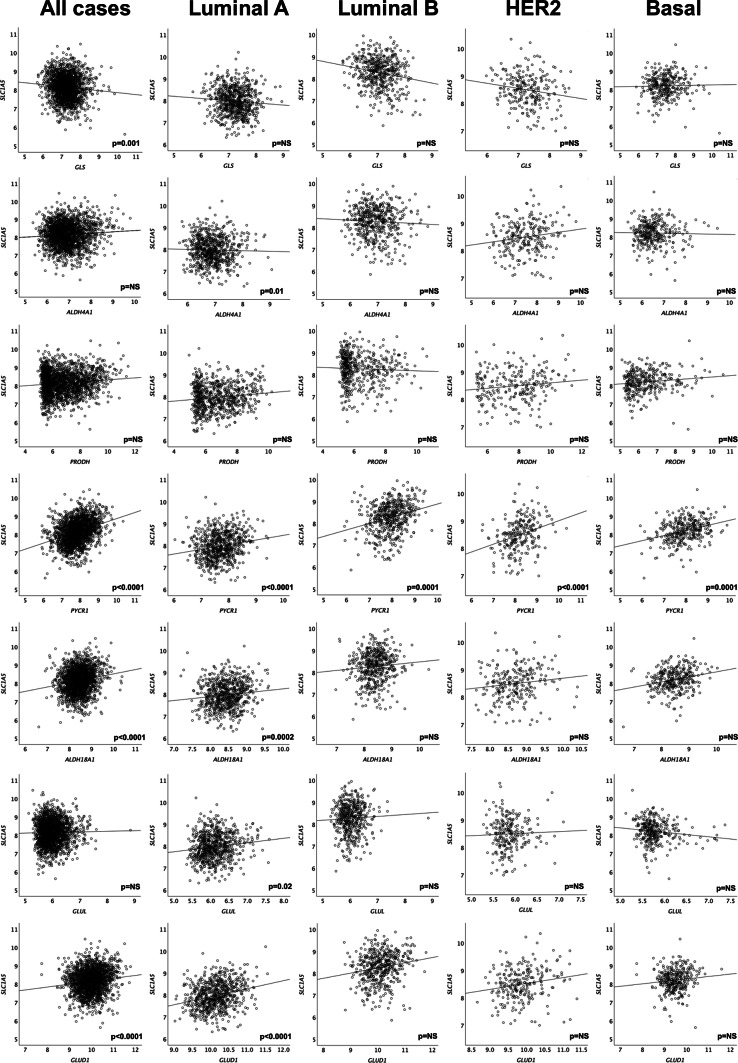
Correlation of *SLC1A5* mRNA expression in invasive breast cancer molecular subtypes with the expression of genes associated with the proline-gluatmine regulatory axis (*GLS*, *ALDH4A1*, *PRODH*, *PYCR1*, *ALDH18A1*, *GLUL*, *GLUD1*) in the METABRIC cohort. NS = not significant.

Regarding associations with glutamine metabolic enzymes, *SLC1A5* expression was positively correlated with enzymes involved in the conversion of glutamine to proline (*GLS*, *PYCR1* and *ALDH18A1*) (P < 0.01, Fig. 2 and Table 2). A positive relationship was also observed with the enzyme *GLUD1*, which catalyses the formation of α-KG from glutamate (P < 0.01). Most enzymes were significantly associated with luminal A tumours (P < 0.05), the only subtype that was positively associated with glutamine synthetase enzyme (GLUL) (P < 0.05). Many amino acid transporters were significantly associated with *SLC1A5* expression, primarily in TN tumours and, to a lesser extent, in luminal tumours (P < 0.05). *SLC7A5* and *SLC38A2* were significantly differentially expressed from *SLC1A5* in all subtypes (P < 0.05).

### Patient outcomes

**Fig. 3:**
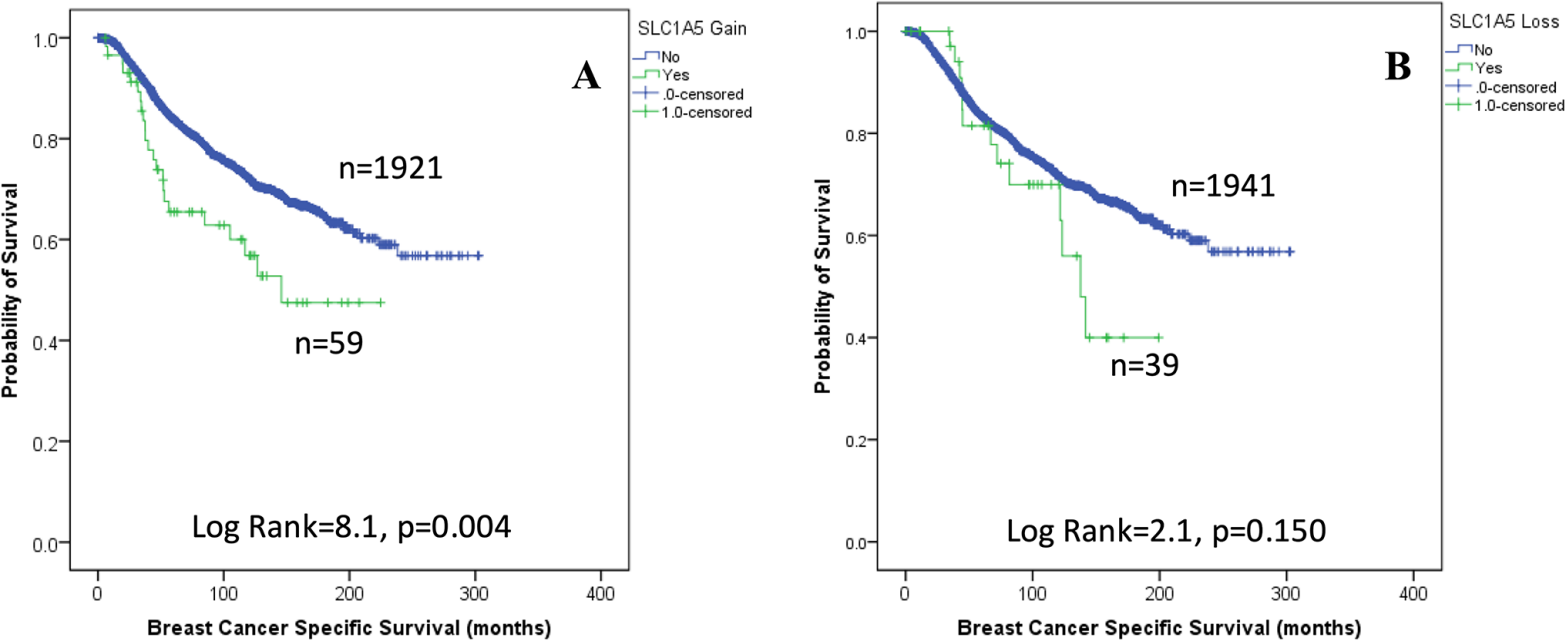
*SLC1A5* copy number aberrations in invasive breast cancer and their relationship with breast cancer-specific survival: (**A**) copy number gain (**B**) copy number loss.

**Fig. 4:**
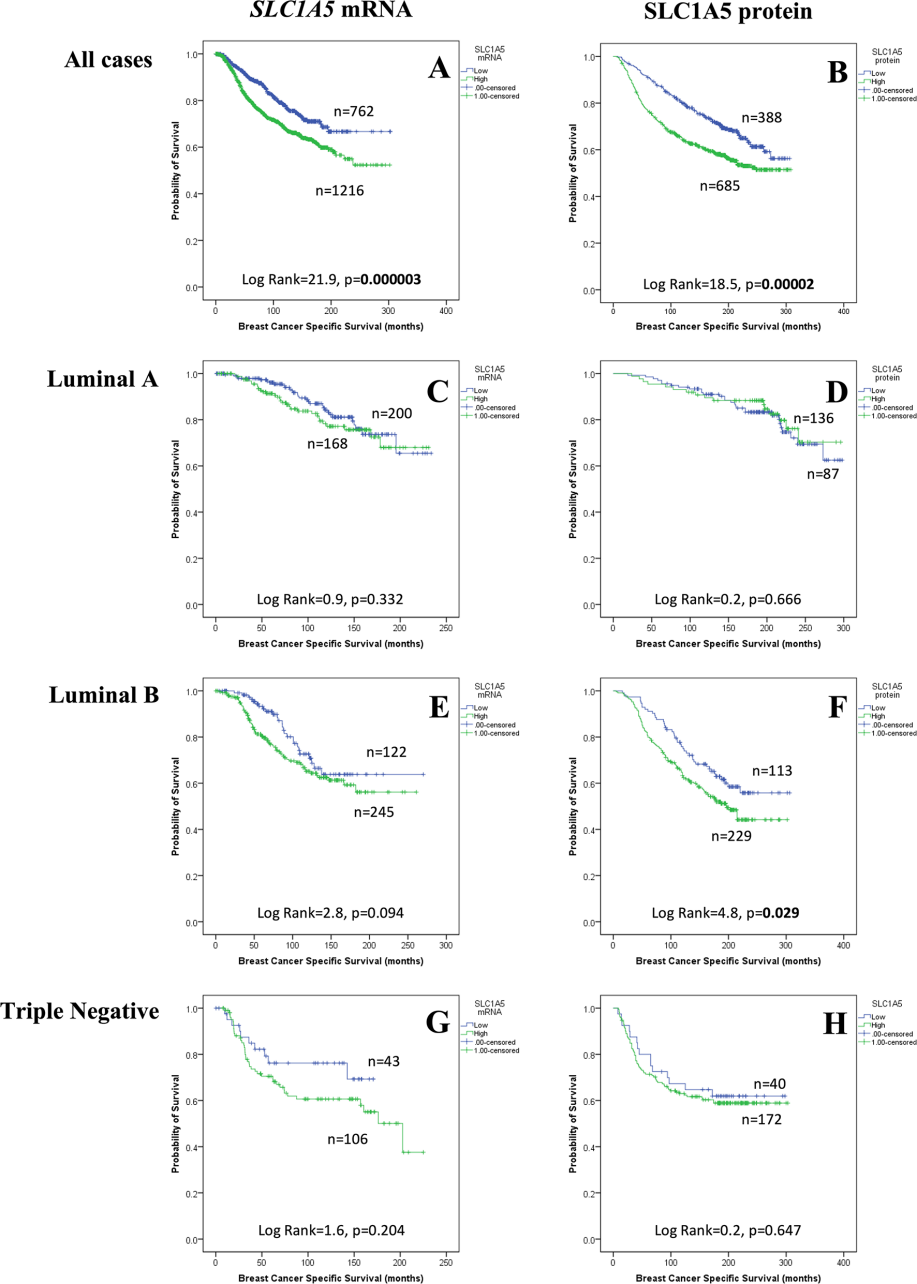
SLC1A5 and breast cancer patient outcome: *SLC1A5* mRNA vs breast cancer specific survival in (**A**) all cases, (**C**) ER + /HER2- Low Proliferation, (**E**) ER + /HER2- High Proliferation, (**G**) Triple Negative tumours and I) HER2 + tumours; SLC1A5 protein vs BCSS in (**B**) all cases, (**D**) ER + /HER2- Low Proliferation, (**F**) ER + /HER2- High Proliferation, (**J**) Triple Negative tumours; (**K**) SLC1A5 protein vs distant metastases free survival in all cases. Green = high; blue = low.

The results demonstrated that CN gain of *SLC1A5*, but not CN loss, was associated with poor patient survival (P = 0.004, Fig. 3). High expression of SLC1A5 *mRNA* was associated with shorter BCSS in all patients (P < 0.001, Fig. 4A), and shorter overall survival (OS) was also observed in the bc-GenExMiner (P < 0.001) but not KM plotter (P > 0.05, Supplementary Fig. 2A-B) datasets. When investigating the expression of *SLC1A5* mRNA in molecular subtypes, high *SLC1A5* mRNA expression tended to be associated with high BCSS in luminal B tumours (P = 0.094, Fig. 4E). There was no association between *SLC1A5 mRNA* and outcomes in patients with other molecular subtypes (P > 0.100, Fig. 4). In the bc-GenExMiner validation dataset, high *SLC1A5* mRNA was predictive of poor OS in patients with luminal A tumours only (P = 0.025, Supplementary Fig. 2C). There was no association between *SLC1A5* mRNA and any of the molecular subtypes according to the KM Plotter dataset (Supplementary Fig. 2).

According to multivariable Cox regression analysis, *SLC1A5 mRNA* remained an independent predictor of BCSS in all patients (P = 0.005, Table 3) but not in any specific subtype (data not shown).

**Table 3:** Multivariate analysis of prognostic variables and SLC1A5 *mRNA* and protein expressions in primary invasive breast cancer.

Parameter	*SLC1A5 mRNA*		SLC1A5 protein	
	Hazard ratio (95% CI)	p-value	Hazard ratio (95% CI)	p-value
SLC1A5	1.55 (1.14–2.12)	**0.005**	1.09 (0.89–1.34)	0.36
Nodal stage	1.72 (1.43–2.05)	**2.7 × 10**^**–9**^	1.89 (1.68–2.12)	**1.4 × 10**^**–27**^
Tumour size	1.45 (1.02–2.06)	**0.03**	1.34 (1.12–1.61)	**0.001**
Tumour grade	1.41 (1.10–1.82)	**0.007**	2.41 (2.03–2.85)	**2.5 × 10**^**–24**^

Significant values are in [bold].

### SLC1A5 protein expression in breast cancers

There was variable expression of the SLC1A5 protein in the membrane of breast tumour cells, ranging from absent to high (Supplementary Fig. 3A–B). Compared to that in invasive breast tumour cells, SLC1A5 protein expression in normal breast epithelium was lower (Supplementary Fig. 3C). High SLC1A5 protein expression (> 40 H-score) was observed in 63% of the tumours. Many patients with high *SLC1A5* mRNA expression also expressed high levels of SLC1A5 protein (correlation coefficient = 0.35, P = 3.3 × 10^−7^).

### Clinicopathological parameters and biological subtypes

Similar associations with *SLC1A5* mRNA were observed with high SLC1A5 protein expression, including larger tumour size, high tumour grade, high pleomorphism, high mitotic count, less tubular formation, poor NPI and the presence of lymphovascular invasion (P < 0.001, Table 4). A significant association was also observed between high SLC1A5 expression and medullary-like tumours (P < 0.001). Regarding BC metastatic sites, high SLC1A5 protein levels were associated with the development of distant metastasis to the bone (P = 0.0006) and liver (P = 0.005, Table 4).

**Table 4:** Clinicopathological associations of SLC1A5 protein expression in primary invasive breast cancer.

SLC1A5 protein				
	Low n (%)	High n (%)	χ2 (p-value)	Adjusted P value
Tumour size				
< 2.0 cm	670 (43.7)	864 (56.3)	57.82 (2.8 × 10^–14^)	** < 0.0001**
≥ 2.0 cm	365 (29.6)	868 (70.4)		
Tumour Grade				
1	304 (68.0)	143 (32.0)	353.77 (1.5 × 10^–77^)	** < 0.0001**
2	459 (44.8)	565 (55.2)		
3	270 (20.9)	1023 (79.1)		
Mitosis				
1	609 (53.8)	524 (46.2)	280.62 (1.1 × 10^–61^)	** < 0.0001**
2	190 (36.2)	355 (63.8)		
3	200 (19.1)	847 (80.9)		
Pleomorphism				
1	33 (71.7)	13 (28.3)	252.04 (1.8 × 10^–55^)	** < 0.0001**
2	507 (56.0)	399 (44.0)		
3	459 (26.2)	1295 (73.8)		
Tubular formation				
1	124 (70.1)	53 (29.9)	123.69 (1.3 × 10^–27^)	** < 0.0001**
2	369 (42.4)	501 (57.6)		
3	507 (30.5)	1153 (69.5)		
Vascular Invasion				
Negative	780 (41.3)	1110 (58.7)	40.77 (1.7 × 10^–10^)	** < 0.0001**
Positive	248 (28.6)	619 (71.4)		
Lymph Node Stage				
1	696 (40.8)	1010 (59.2)	28.73 (5.7 × 10^–7^)	** < 0.0001**
2	275 (34.1)	531 (65.9)		
3	62 (24.9)	187 (75.1)		
Nottingham Prognostic Index				
Good	505 (56.4)	390 (43.6)	219.55 (2.1 × 10^–48^)	** < 0.0001**
Moderate	438 (30.8)	985 (69.2)		
Poor	91 (20.4)	354 (79.6)		
Biological Subtypes				
ER + /HER2- Low Proliferation	704 (49.8)	710 (50.2)	231.2 (7.5 × 10^–50^)	** < 0.0001**
ER + /HER2- High Proliferation	99 (22.3)	344 (77.7)		
Triple Negative	77 (18.0)	351 (82.0)		
HER2 +	61 (22.5)	210 (77.5)		
Histological type				
Ductal (including mixed)	836 (35.2)	1536 (64.8)	87.18 (2.6 × 10^–17^)	** < 0.0001**
Lobular	103 (47.7)	113 (52.3)		
Medullary	3 (7.5)	37 (92.5)		
Miscellaneous	9 (50.0)	9 (50.0)		
Special type	83 (70.9)	34 (29.1)		
Tubular	1 (33.3)	2 (66.7)		
Site of distant metastasis				
Brain				
No	973 (55.9)	767 (44.1)	1.73 (0.18)	0.54
Yes	45 (48.9)	47 (51.1)		
Lung				
No	929 (55.8)	737 (44.2)	0.282 (0.59)	1.18
Yes	89 (53.6)	77 (46.4)		
Bone				
No	848 (57.8)	620 (42.2)	14.45 (0.0001)	**0.0006**
Yes	170 (46.7)	194 (53.3)		
Liver				
No	903 (57.1)	679 (42.9)	10.73 (0.001)	**0.005**
Yes	115 (46.0)	135 (54.0)		

Significant values are in [bold].

High SLC1A5 protein expression was significantly associated with ER-negative, PR-negative and HER2-positive tumours (P < 0.001, Table 1). Additionally, SLC1A5 protein expression was associated with TN tumours (P < 0.001, Table 1). SLC1A5 protein expression in the IHC-defined molecular subtypes was significantly lower in the luminal A tumours than in the other subtypes (P < 0.001, Table 4).

### Regulatory and amino acid transporter proteins

The associations of SLC1A5 protein expression with other proteins were also examined (Table 5). SLC1A5 protein was significantly expressed in breast tumours with high Ki67 (P < 0.001) and MYC (P = 0.02) expression. PIK3CA was also significantly expressed in breast tumours with high SLC1A5 expression (P = 0.005). Interestingly, high SLC1A5 protein expression was significantly associated with all enzymes involved in the glutamine-proline regulatory axis, including enzymes that convert glutamine to proline (GLS, PYCR1 and ALDH18A1) (P < 0.001) and enzymes that catalyse proline to glutamine (PRODH and ALDH4A1) (P < 0.05). Apart from SLC7A8, all the following amino acid transporters, SLC7A5, SLC3A2, SLC7A11 and SLC38A2, were significantly expressed in breast tumours with high SLC1A5 expression (P < 0.01).

**Table 5:** Association of SLC1A5 protein and other biological markers in primary invasive breast cancer.

	SLC1A5 protein			
	Low, n (%)	High, n (%)	χ2 (p-value)	Adjustedp-value
c-MYC				
Negative	292 (35.4)	534 (64.6)	7.27 (0.007)	**0.02**
Positive	41 (24.6)	126 (75.4)		
Ki67				
Negative	184 (55.1)	150 (44.9)	89.8 (2.5 × 10^–21^)	** < 0.0001**
Positive	184 (25.3)	544 (74.7)		
PIK3CA				
Negative	97 (43.7)	125 (56.3)	10.68 (0.001)	**0.005**
Positive	227 (31.7)	488 (68.3)		
GLUD1				
Negative	292 (33.7)	575 (66.3)	1.78 (0.18)	0.36
Positive	128 (37.8)	211 (62.2)		
GLS				
Negative	216 (39.2)	335 (60.8)	28.79 (8.0 × 10^–8^)	** < 0.0001**
Positive	92 (22.8)	312 (77.2)		
PYCR1				
Negative	171 (39.7)	260 (60.3)	29.89 (4.5 × 10^–8^)	** < 0.0001**
Positive	80 (21.7)	289 (78.3)		
ALDH4A1				
Negative	166 (38.2)	268 (61.8)	10.39 (0.001)	**0.006**
Positive	121 (27.9)	312 (72.1)		
ALDH18A1				
Negative	183 (40.1)	273 (59.9)	20.59 (0.000006)	** < 0.0001**
Positive	112 (25.8)	322 (74.2)		
PRODH				
Negative	240 (33.8)	471 (66.2)	6.30 (0.01)	**0.03**
Positive	46 (24.2)	144 (75.8)		
SLC7A5				
Negative	825 (41.3)	1174 (58.7)	161.7 (4.7 × 10^–37^)	** < 0.0001**
Positive	39 (9.0)	394 (91.0)		
SLC3A2				
Negative	423 (41.3)	601 (58.7)	32.40 (1.2 × 10^–8^)	** < 0.0001**
Positive	308 (29.4)	741 (70.6)		
SLC38A2				
Negative	487 (34.7)	917 (65.3)	12.92 (0.0003)	**0.002**
Positive	27 (19.6)	111 (80.4)		
SLC7A8				
Negative	430 (33.4)	856 (66.6)	1.50 (0.22)	0.44
Positive	47 (28.7)	117 (71.3)		
SLC7A11				
Negative	430 (38.5)	686 (61.5)	27.8 (1.3 × 10^–7^)	** < 0.0001**
Positive	197 (26.7)	541 (73.3)		

Significant values are in [bold].

### Patient outcomes

The results demonstrated that high SLC1A5 protein expression was associated with shorter BCSS in all patients (P < 0.001; Fig. 4B). When investigating the expression of SLC1A5 protein in biological subtypes, high expression was only predictive of shorter BCSS in luminal B tumours (P < 0.05, Fig. 4F). There was no association between SLC1A5 protein and outcome in luminal A (Fig. 4D), TN (Fig. 4H) or HER2 + (Fig. 4J) tumours. According to multivariable analysis, SLC1A5 protein and other clinicopathological parameters were not significantly associated with BCSS (P > 0.05, Supplementary Table 3).

### SLC1A5 is required for cell proliferation and glutamine uptake

**Fig. 5:**
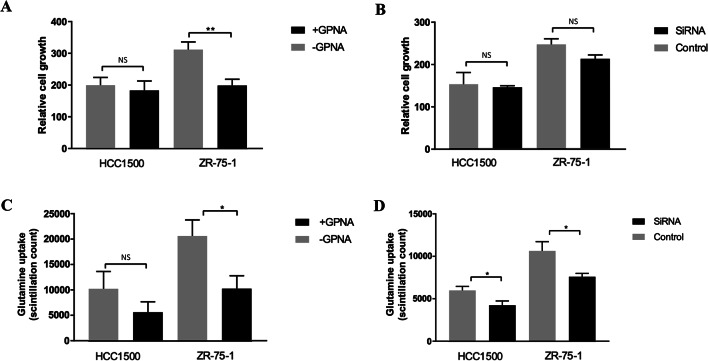
The effect of SLC1A5 inhibition on the growth (**A**–**B**) and glutamine uptake (C-D) of luminal breast cancer cell lines using the GPNA inhibitor (**A**, **C**) and *SLC1A5* mRNA knockdown (**B**, **D**) using siRNA. NS = not significant, *p < 0.05, **p < 0.01.

SLC1A5 protein expression in a normal basal mammary cell line (MCF10) and a panel of luminal BC cell lines was greater in ZR-75-1 and HCC1500 cells than in the other cells analysed (Supplementary Fig. 4A). *SLC1A5* knockdown and SLC1A5 inhibition were confirmed in ZR-75-1 and HCC1500 cells by western blotting (Supplementary Fig. 4B-C). Cell proliferation was significantly decreased by GPNA inhibition in ZR-75-1 cells but not in HCC1500 cells (Fig. 5A). However, targeted knockdown of *SLC1A5* did not significantly impair the proliferation of ZR-75-1 cells or HCC1500 cells (Fig. 5B). GPNA reduced glutamine uptake in ZR-75-1 cells but not in HCC1500 cells (Fig. 5C). Glutamine uptake was lower in both ZR-75-1 and HCC1500 cells transfected with siRNA targeting *SLC1A5* than in control cells (Fig. 5D).

## Discussion

ER + /luminal tumours, which constitute approximately 75% of BCs^39,40^, remain a heterogeneous group in terms of molecular biology and patient outcomes. Therefore, there is a clear need for improved understanding of the biology of the luminal class of BC, with subsequent translation into more effective methods for the diagnosis and management of this most common form of BC.

Metabolic reprogramming in cancer plays a vital role in the provision of supplementary elements, including nutrients and energy, which are essential for cellular growth. It has been reported that tumour cells rely on glutamine metabolism and become “addicted” to this amino acid for sustained proliferation/survival. Studies that address the prognostic significance of the key Gln transporter SLC1A5 in BC and its potential influence on Gln metabolism in different subtypes remain limited, particularly in luminal BC. We therefore investigated *SLC1A5* mRNA and SLC1A5 protein expression in a large number of breast tumours to better understand the potential role of this important transporter of Gln in BC and its molecular subtypes, particularly in luminal ER + disease.

In this study, we have shown that SLC1A5, which is a primary transporter for Gln uptake, is highly expressed in a subset of ER + tumours that have high proliferation, i.e., luminal B tumours, and is related to poor patient outcome in this group. The high expression of SLC1A5 in luminal B tumours is perhaps not surprising because these tumours have greater demands for nutrients and energy, which are essential for cell survival and proliferation. SLC1A5 has previously been shown to be a poor prognostic factor in BC^41^. Similarly, Jeon et al*.* showed that high SLC1A5 was associated with shorter disease-free survival in patients with ER + BC, but neither investigated the molecular subtypes^15^. We further demonstrated that the association of SLC1A5 with patient outcome occurs only in luminal B and not luminal A tumours.

We confirmed that the SLC1A5 protein is expressed in TNBC and HER2 + cells, which is in accordance with the findings of Van Geldermalsen et al*.*^8^, and we additionally showed that *SLC1A5* mRNA is also highly expressed in these subtypes^10^, confirming the possible role of the transcription and translation of this amino acid transporter in driving the uptake of Gln, which is required for proliferation in these highly proliferative subtypes of BC. However, in both subtypes, there was no association between *SLC1A5* mRNA or protein expression and patient outcome.

Previous studies have raised awareness and revealed the importance of the proline-glutamine (Pro-Gln) regulatory axis in BC as part of tumour metabolism in addition to glycolysis according to different BC subtypes^42^, mainly focusing on either Gln or Pro metabolism only. The acquisition of glutamine via SLC1A5 is undoubtedly important, but proline metabolism may be an alternative source of glutamine. The coexpression of genes encoding Pro-Gln enzymes with SLC1A5 suggested that luminal A tumours might be partly glutamine independent rather than relying on uptake via *SLC1A5,* as in the basal HCC1806 cell line^8^. We therefore compared the gene expression of Pro-Gln enzymes with that of *SLC1A5* and showed highly variable expression of this regulatory axis across molecular subtypes. Luminal A tumours had the highest number of correlates focused on a positive association with enzymes involved in the conversion of Pro to Gln, whereas those genes involved in the conversion of Gln to Pro were primarily downregulated. The correlation between *SLC1A5* and *GLUD1*, which is involved in the conversion of Gln to alpha-ketoglutarate for the TCA cycle and subsequent gluconeogenesis, in luminal A tumours suggests that Gln is utilised for this process. Only *PCYR1* was associated with *SLC1A5* in luminal B tumours, suggesting that the primary source of Gln in these tumours is via uptake rather than neosynthesis. This might explain why MCF-7 luminal A cells are not affected by Gln deprivation when SLC1A5 is blocked with GPNA^8^.

Previous studies have shown that SLC1A5 is regulated by other proteins, including the tumour oncogene MYC, which induces SLC1A5. In the current study, we sought to understand the relationship between SLC1A5 and other regulatory proteins in terms of both mRNA and protein expression. We observed a positive correlation between SLC1A5 and MYC at the protein level but not at the mRNA level. MYC also induces apoptosis via ATF4 upon glutamine deprivation, and we observed a positive correlation between *ATF4* and *SLC1A5* gene expression, in line with expectations. We have recently investigated ATF4 protein expression in invasive BC and its coexpression with SLC1A5 protein is associated with poor patient outcome in ER + tumours^42^. Chemotherapy treatment in BC promotes the degradation of SLC1A5 via RNF5 ubiquitination, leading to mTOR inactivation^15^, although we did not observe any association between the gene expression of *SLC1A5* and *RNF5* or its pseudogene *RNFP1*.

There are more than 24 amino acid transporters, and we further investigated whether SLC1A5 expression was associated with any of the key transporters. SLC7A5 functionally couples with SLC1A5 to allow cellular influx and efflux of Gln. The coexpression of SLC1A5 and SLC7A5 in all BC subtypes suggested that they play a key role in Gln transport. Indeed, SLC7A5 has previously been incorporated into the Mammostrat® risk test used to stratify BC patients treated with tamoxifen^43^. SLC1A5 also requires SLC7A11 for functional coupling of glutamine efflux and cystine entry, which in turn is converted to cysteine, which rules SLC1A5-mediated glutamine entry, although in our study, SLC7A11 was associated with luminal A tumours^44^. The coexpression of SLC1A5, SLC7A11 and SLC7A5 proteins has yet to be determined in BC and is therefore important for understanding the potential transport of Gln in and out of tumour cells.

With the increasing number of treatment strategies available for BC patients, it is important that effective strategies that can support the personalisation of care and allow tailored treatment planning appropriate for patients’ tumour biology, both to maximise treatment benefit and to avoid the adverse effects associated with over- or undertreatment, emerge. This would minimise harmful side effects to patients and reduce treatment costs by focusing expensive and valuable resources on those who would optimally benefit from the new generation of targeted molecular therapies. For instance, it has been proposed that one of the mechanistic actions of tamoxifen involves the suppression of glutamine uptake and the induction of apoptosis^45^. SLC1A5 has also been linked to endocrine therapy resistance in luminal BC. Inhibiting or depleting SLC1A5 in these cells has been shown to increase sensitivity to tamoxifen and decrease proliferation in aromatase inhibitor-resistant cells^46,47^. Additionally, high SLC1A5 expression in clinical samples correlates with endocrine therapy resistance and worse patient outcomes in luminal BC^46^.

Blocking SLC1A5 using the small molecule inhibitor GPNA inhibited Gln uptake and subsequent tumor growth in basal-like TNBC but not in luminal A tumours using MCF-7 cells as an in vitro model^8^. Although the consequences of blocking SLC1A5 in luminal B tumours remain undetermined, our data suggest that SLC1A5 could be used as a target in luminal B tumours to reduce Gln uptake and thus cell proliferation and growth. In addition to GPNA, 2-amino-4-bis (aryloxybenzyl) aminobutanoic acids have recently been identified as novel inhibitors of Gln uptake via SLC1A5 ^48^. Evaluation of these and other inhibitors is therefore warranted in luminal B BC.

Therefore, we believe that continued refinement of the understanding of the biological diversity of BC, particularly the luminal B subtype, with linked development of classification strategies suitable for routine clinical use is essential to achieve a personalised approach to BC management. Further assessment of the metabolic pathways associated with glutamine and its uptake is therefore essential in the luminal B subtype and other subtypes, including HER2 + tumours.

## Electronic supplementary material

Below is the link to the electronic supplementary material.

Supplementary Material 1

## Data Availability

The data that support the findings of this study are available from the corresponding author upon reasonable request.
