# Supplementary material for: SLC1A5 is a key regulator of glutamine metabolism and a prognostic marker for aggressive luminal breast cancer
Source: Sci Rep. 2025 Jan 22;15:2805. doi: 10.1038/s41598-025-87292-1 (PMC11754656; doi:10.1038/s41598-025-87292-1)
Supplement: Supplementary file 1 — Supplementary Material 1 [file 41598_2025_87292_MOESM1_ESM.pdf]

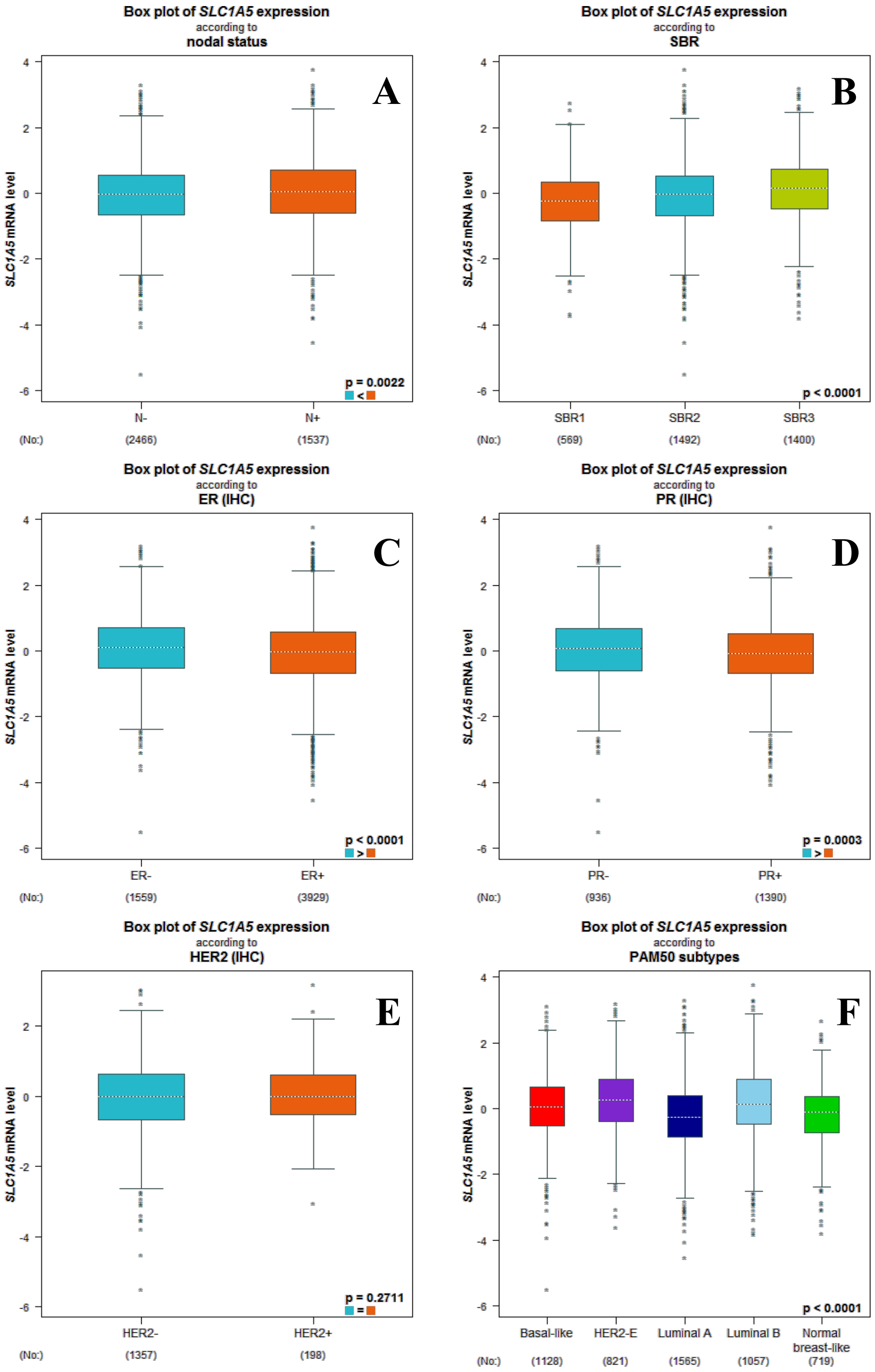


**Supplementary Figure 1**: *SLC1A5* gene expression and its association, using Breast Cancer Gene-Expression Miner, with: A) lymph node stage, B) tumour grade, C) ER status, D) HER2 status, E) PAM50 subtypes.

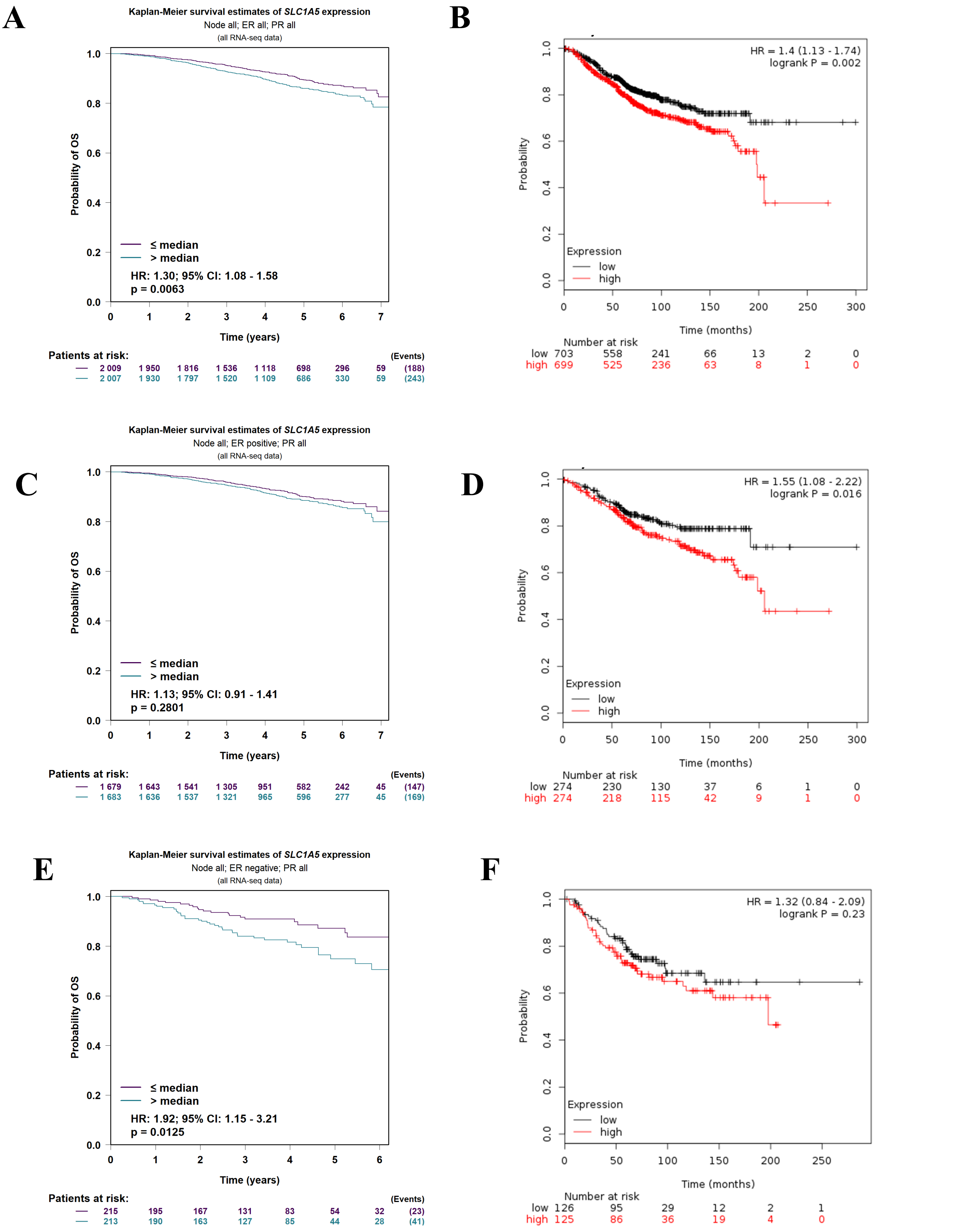


**Supplementary Figure 2.** *SLC1A5* mRNA and breast cancer patient outcome using Breast Cancer Gene-Expression Miner (A, C, E) and KM Plotter (B, D, F) in unselected cases (A-B), ER+ BC (C-D) and ER- BC (E-F).

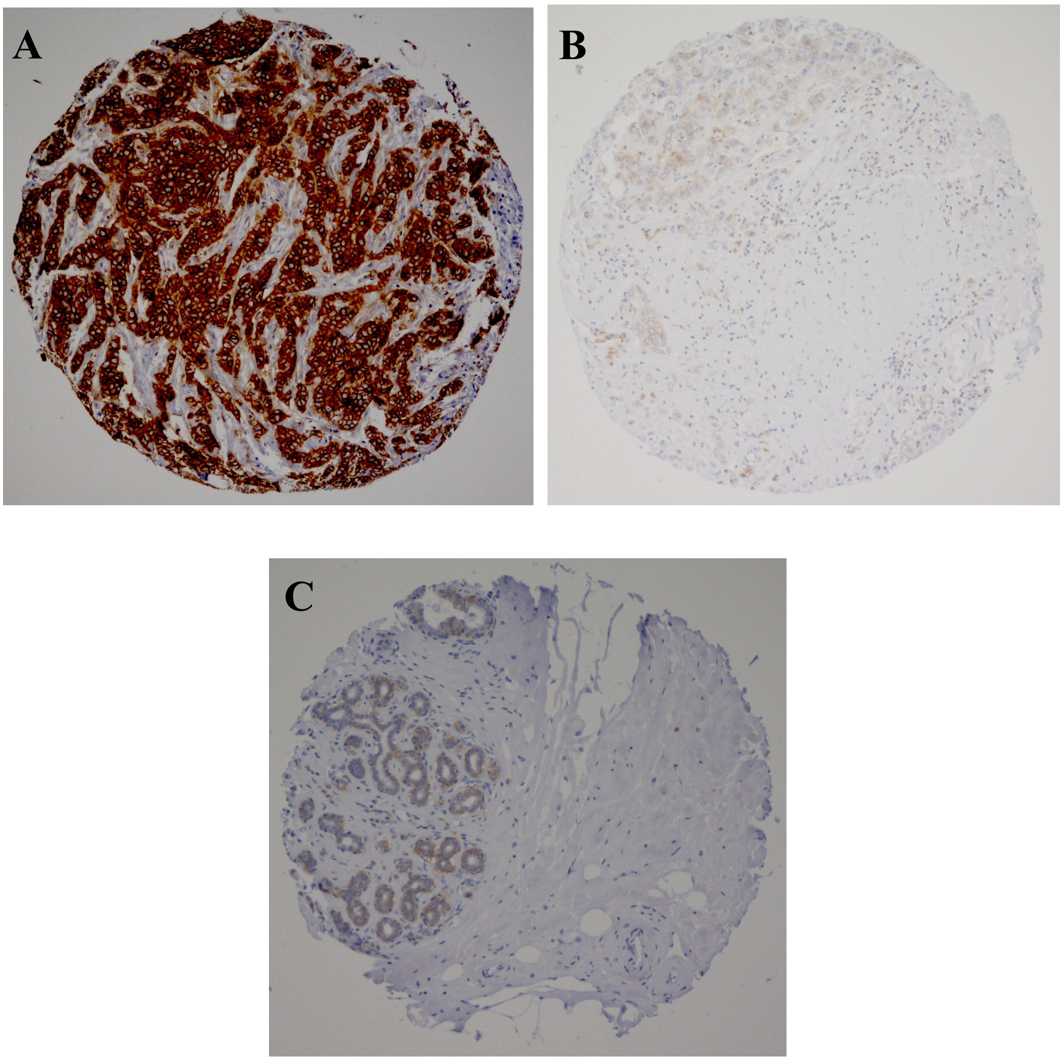
A
B
C

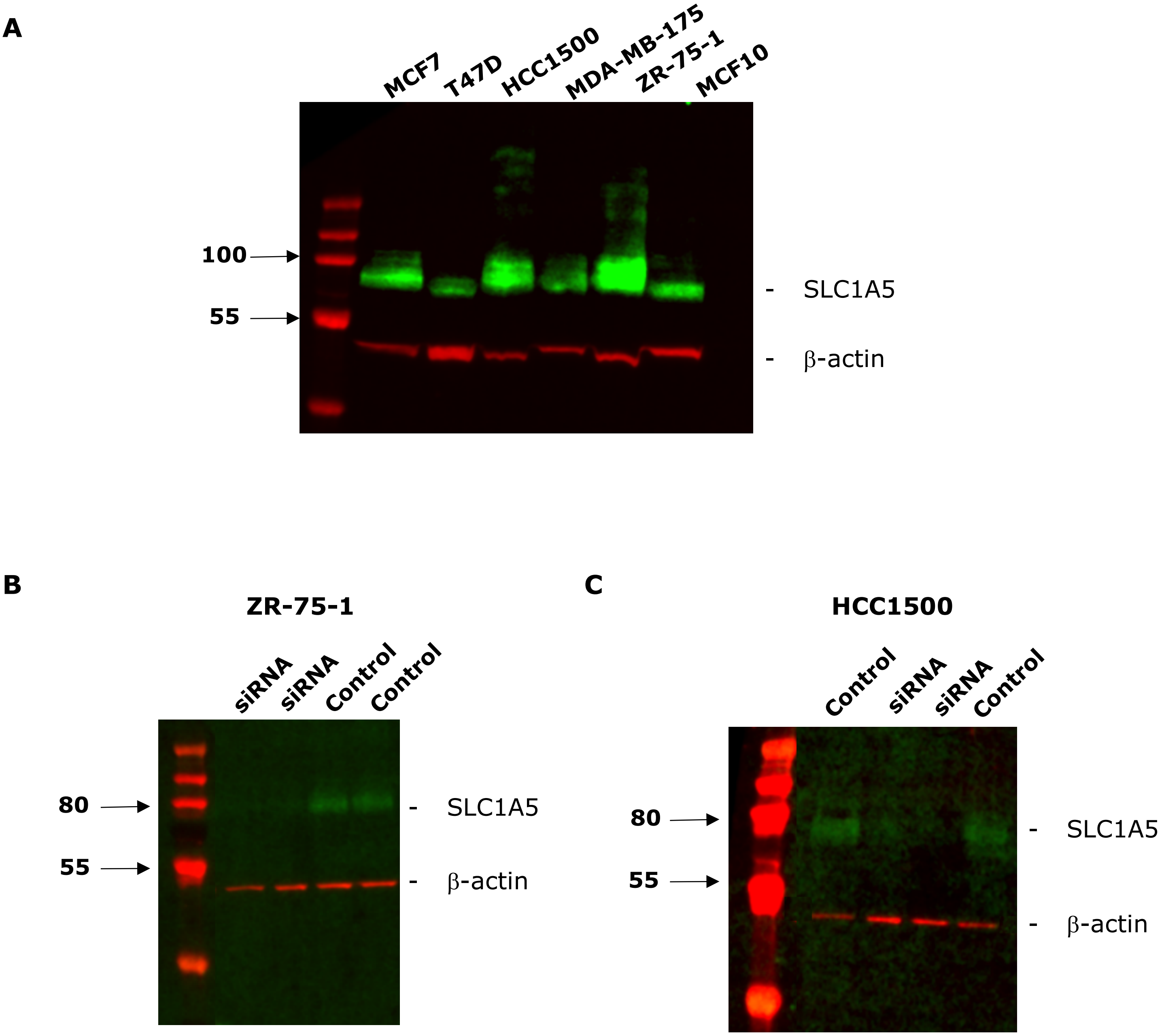
A
MCF7
T47D
HCC1500
MDA-MB-175
ZR-75-1
MCF10
100
55
SLC1A5
β-actin
B
ZR-75-1
siRNA
siRNA
Control
Control
80
55
SLC1A5
β-actin
C
HCC1500
Control
siRNA
siRNA
Control
80
55
SLC1A5
β-actin
